# The whole is greater than the sum of its parts: effect of implementing an ABS consultation service in the ABS program on antibiotic consumption at a tertiary military hospital

**DOI:** 10.3205/dgkh000540

**Published:** 2025-03-10

**Authors:** David Zeidler, Gwendolyn Scheumann, Claudia Baessler, Manuel Döhla, Dominic Preuß, Dominic Rauschning

**Affiliations:** 1 Department of Pharmacy, Bundeswehr Central Hospital Koblenz, Germany; 2 Department IB of Internal Medicine, Division of Infectious Diseases, Bundeswehr Central Hospital Koblenz, Germany; 3 Department of Medical Microbiology and Hospital Hygiene, Bundeswehr Central Hospital Koblenz, Germany

**Keywords:** antibiotic stewardship, antibiotics, antibacterial drug resistance, multidisciplinary care team

## Abstract

**Background:**

Infections with multidrug resistant (MDR) pathogens represent an enormous challenge for the healthcare system. By implementing the principles of Antibiotic Stewardship (ABS), the rational use of antibiotics is promoted to counteract the increasing development of resistance on the one hand and to ensure adequate treatment for patients on the other.

**Methods:**

An interdisciplinary ABS team has therefore been set up at the Bundeswehr Central Hospital and is available to medical staff every working day as part of a consultation and ward-rounds service. The work of the ABS team primarily serves to advise on the treatment, diagnosis and prevention of infectious diseases. It also intends to provide knowledge during the training of young medical officers in order to promote the rational use of antibiotics even outside the Bundeswehr Central Hospital.

**Results:**

Since the implementation of the consultation and ward-rounds service, antibiotic consumption at the Bundeswehr Central Hospital has been reduced by 25%. In this context, the halving of carbapenem consumption is particularly noteworthy. Furthermore, the rising number of consultation requests shows an increasing need for the service and acts as a marker for ABS awareness.

**Conclusion:**

The reduction in antibiotic consumption is not solely due to the mentioned service, but is also the result of interdisciplinary ABS measures.

## Zusammenfassung

**Hintergrund:**

Infektionen mit multiresistenten Erregern (MRE) stellen für das Gesundheitssystem eine enorme Herausforderung dar. Durch Implementierung der Prinzipien von Antibiotic Stewardship (ABS) wird der rationale Einsatz von Antibiotika gefördert, um einerseits einer zunehmenden Resistenzentwicklung entgegenzuwirken und andererseits für Patientinnen und Patienten eine adäquate Therapie zu gewährleisten.

**Methoden:**

Im Bundeswehrzentralkrankenhaus (BwZKrhs) wurde daher ein interdisziplinäres ABS-Team gegründet, das dem ärztlichen Personal im Rahmen eines Konsil- und Visitendienstes arbeitstäglich zur Verfügung steht. Neben Empfehlungen zur Behandlung, Diagnostik und Prävention von Infektionskrankheiten dient die Arbeit des ABS-Teams auch dem Wissenstransfer, um den rationalen Einsatz von Antibiotika im Rahmen der Ausbildung junger Sanitätsoffiziere perspektivisch auch außerhalb des BwZKrhs zu fördern.

**Ergebnisse:**

Seit der Implementierung des Visiten- und Konsildienstes konnte die Antibiotikaverbrauchsdichte des BwZKrhs um 25% reduziert werden. In diesem Zusammenhang ist insbesondere die Halbierung des Carbapenemverbrauchs hervorzuheben. Weiterhin zeigt die steigende Zahl der Konsilanforderungen einen zunehmenden Bedarf für die genannte Dienstleistung und dient ebenfalls als Marker für das Bewusstsein für ABS.

**Schlussfolgerung:**

Die Reduktion der Antibiotikaverbrauchsdichte ist dabei nicht allein auf den Visiten- und Konsildienst zurückzuführen, sondern das Resultat eines interdisziplinären ABS-Maßnahmenpakets.

## Background

The increasing resistance of pathogens worldwide is a serious challenge for the healthcare system. As early as 2014, a British modelling study warned of 10 million deaths in 2050 due to resistance to antimicrobial substances [1]. After analyzing real data, Murray et al. [2] indicated that in 2019, around 6.3 million deaths worldwide occurred in connection with resistant bacteria or were directly attributed to them. 

Figures from the European Centre for Disease Prevention and Control (ECDC) clearly show that the number of infections caused by pathogens with multidrug resistance (MDR) is also increasing in Europe. According to a report from 2022, around 35,000 Europeans died each year between 2016 and 2020 as a result of infections with resistant pathogens. The agency estimates that 70% of these infections were acquired nosocomially [3]. 

The progressive loss of efficacy of antibiotics is further exacerbated by the lack of development for new anti-infective agents. A recent WHO report criticizes a lack of innovation and investment in the field of antibiotic development [4]. In addition, the increasingly frequent occurrence of supply shortages continues to intensify the precarious situation [5], [6]. 

Of particular concern is the more frequent occurrence of *Klebsiella* *pneumoniae*, *Pseudomonas* *aeruginosa* and *Acinetobacter* *baumannii*, which are showing increasing carbapenem resistance. Due to the resistance spectrum, only extremely limited treatment options are available for the pathogens mentioned, which are therefore again included in the 2024 update of the WHO bacterial priority pathogens list [7]. In order to counter such complicated infections with effective treatment regimes, the necessary skills must be taught and acquired in hospitals. The same applies to preventive measures to keep the prevalence of MDR in Germany low.

To counteract the triad of resistance-related loss of efficacy, insufficient development of new anti-infective agents, and limited availability of existing antibiotic drugs, measures are needed to ensure adequate prescribing practices for the scarce and valuable resource of antibiotics. In this regard, antibiotic stewardship (ABS) strategies are used to promote the rational use of antibiotics.

The aim of this retrospective evaluation was to examine the acceptance of the ABS consultation and ward-rounds service at the Bundeswehr Central Hospital Koblenz (BundeswehrZentralkrankenhaus, BwZKrhs) in Koblenz, Germany. The BwZKrhs is a tertiary military hospital with 364 beds and 19 departments and focuses on trauma, cardiovascular, and oncological care. This study also aimed to evaluate the extent to which ABS measures lead to short-term changes in the consumption of antibiotics.

## Methods

In 2012, an interdisciplinary ABS commission was set up at the BwZKrhs on the foundation of national legislation [8] and the German S3 guideline “Strategies for ensuring the rational use of antibiotics in hospitals” [9] in order to further improve the anti-infective treatment of patients. An ABS team was implemented by the ABS commission in the 2^nd^ quarter of 2021, which is made up of specialists from the fields of infectiology, clinical pharmacy, microbiology and hospital hygiene in accordance with the above-mentioned guideline. The task of the ABS team is to advise clinical staff on the prevention, diagnosis, and treatment of infections and to implement measures for the rational use of anti-infectives.

### ABS consultation service

Every working day, all physicians at the hospital have the opportunity to submit requests for the optimization of anti-infective therapy in the form of a consultation via the hospital information system (HIS), which can be viewed by the ABS team members. Initially, the relevant microbiological findings are analysed, which are automatically made available in the patient files by the microbiological laboratory. At the same time, other diagnostics that have already been carried out are reviewed by ABS team members. The patient is usually visited in person by clinical infectiologists and, if necessary, other ABS team members. Specific recommendations for optimizing anti-infective therapy and further diagnostics are discussed in person with the treating physician and written down in the HIS consultation form.

If necessary, the ABS team asks for a re-consultation to monitor progress. Since mid-2023, ABS-trained nurses for hygiene and infection prevention in hospital hygiene have increasingly taken on the task of following up on the recommendations made in consultations. These reviews allow questions or ambiguities to be clarified that often lead to missing or delayed implementation. If a *Staphylococcus* *aureus* bloodstream infection is detected, ABS consults are implemented automatically by staff from the microbiological laboratory. 

### ABS ward rounds

A weekly ABS ward-round in the intensive care units (ICUs) of the anesthesiology and the internal medicine departments at the BwZKrhs Koblenz was scheduled in order to optimize the anti-infective treatment of critically ill patients and to prevent the unnecessary use of broad-spectrum antibiotics. 

By registering the patients to be discussed via a HIS consultation request by the day before the ward-round, the ABS team can be adequately prepared for the usually complex patient cases. The ABS visit is carried out by the attending multi-professional ABS team to ensure an interdisciplinary case discussion. Following the presentation of the patient by a physician of the treating ICU, therapy recommendations for de-escalation, dose adjustment, initiation of targeted therapy, further diagnostics, or duration of therapy are discussed and decided by consensus. Implementation is carried out by the treating staff directly. The result of the ward-round is documented by the ABS team on the HIS consultation form.

### External benchmark

BwZKrhs Koblenz has been taking part in a national “ADKA-if-DGI” project to determine antibiotic consumption in hospitals since 2020 [10]. For this purpose, the respective consumption of antibiotics per ward is determined using the transactions in the hospital pharmacy’s logistics software. This logistical data is transmitted to the project on a quarterly basis together with the generated case numbers and patient days. The evaluation is carried out as consumption density in recommended daily doses (RDD) per 100 patient days. Due to its therapeutic reference to patient days, the RDD provide a more accurate approximation of the actually used doses than defined daily doses (DDD) [11]. The consumption densities are not reported for individual active substances, but for 13 defined groups of antibiotics.

The voluntary participation of 329 hospitals throughout Germany enables a comparison of consumption densities with hospitals with a comparable number of beds, thus providing an external benchmark.

### Statistics

The number of ABS consultation requests for the period from the 2^nd^ quarter 2021 to the end of 2023 was extracted from the HIS. Additionally, the antibiotic consumption data for the same period was used to establish comparisons and correlations. 

Statistical calculations were performed using Stata 15.1 IC (Stata Corp, Texas, USA). Correlations of metrically scaled variables were checked using Pearson’s product moment correlation and the correlation coefficient r was reported. Cuzick’s test for trends was used to test the development of antibiotic consumption density. In the tests performed, a *p*<0.05 was considered statistically significant. The antibiotic consumption density for different drug groups and time periods was compared and tested for statistical significance using the freely available MedCalc calculator for the comparison of two rates (https://www.medcalc.org/calc/index.php, MedCalc Software, Ostend, Belgium). With required 95% confidence intervals (95% CI), *p*-values <0.05 were also considered statistically significant.

## Results

### Development of antibiotic consumption density

**Figure 1:**
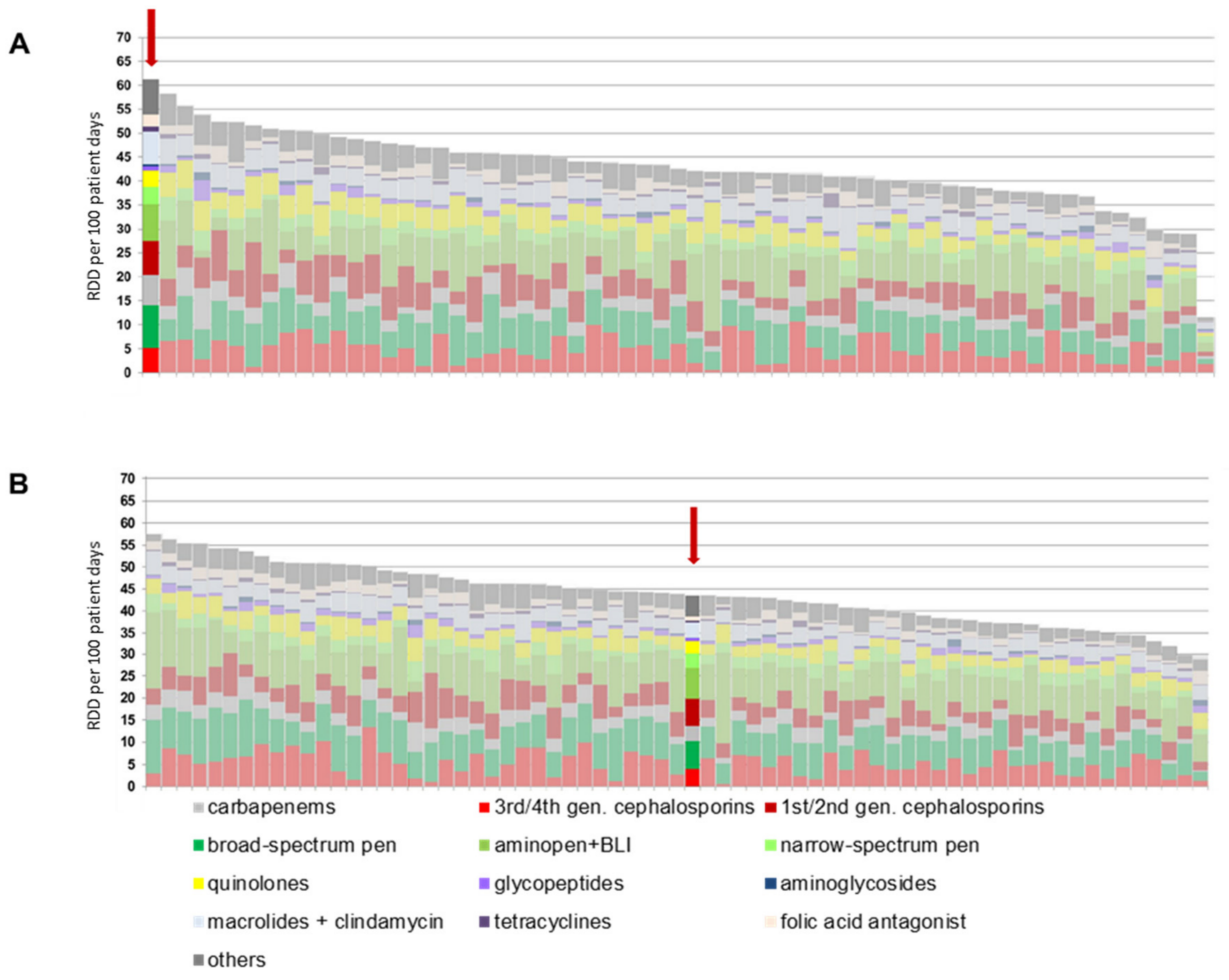
Antibiotic consumption of the hospitals participating in the national ADKA-if-DGI project for the years 2020 (A) and 2022 (B); the column for the Bundeswehr Central Hospital is highlighted in color and marked with a red arrow. RDD: recommended daily dose; gen.: generation; BLI: beta-lactamase inhibitor.

Before the introduction of the ABS consultation service, BwZKrhs Koblenz had the highest antibiotic consumption density in the national project within its peer group of hospitals with 400 to 800 beds (Figure 1A (Fig. 1)). In the nationwide comparison of the participating hospitals, BwZKrhs also had the eleventh-highest consumption density of the 329 participating hospitals. The high consumption of carbapenems was remarkable.

### Overall internal trend

**Figure 2:**
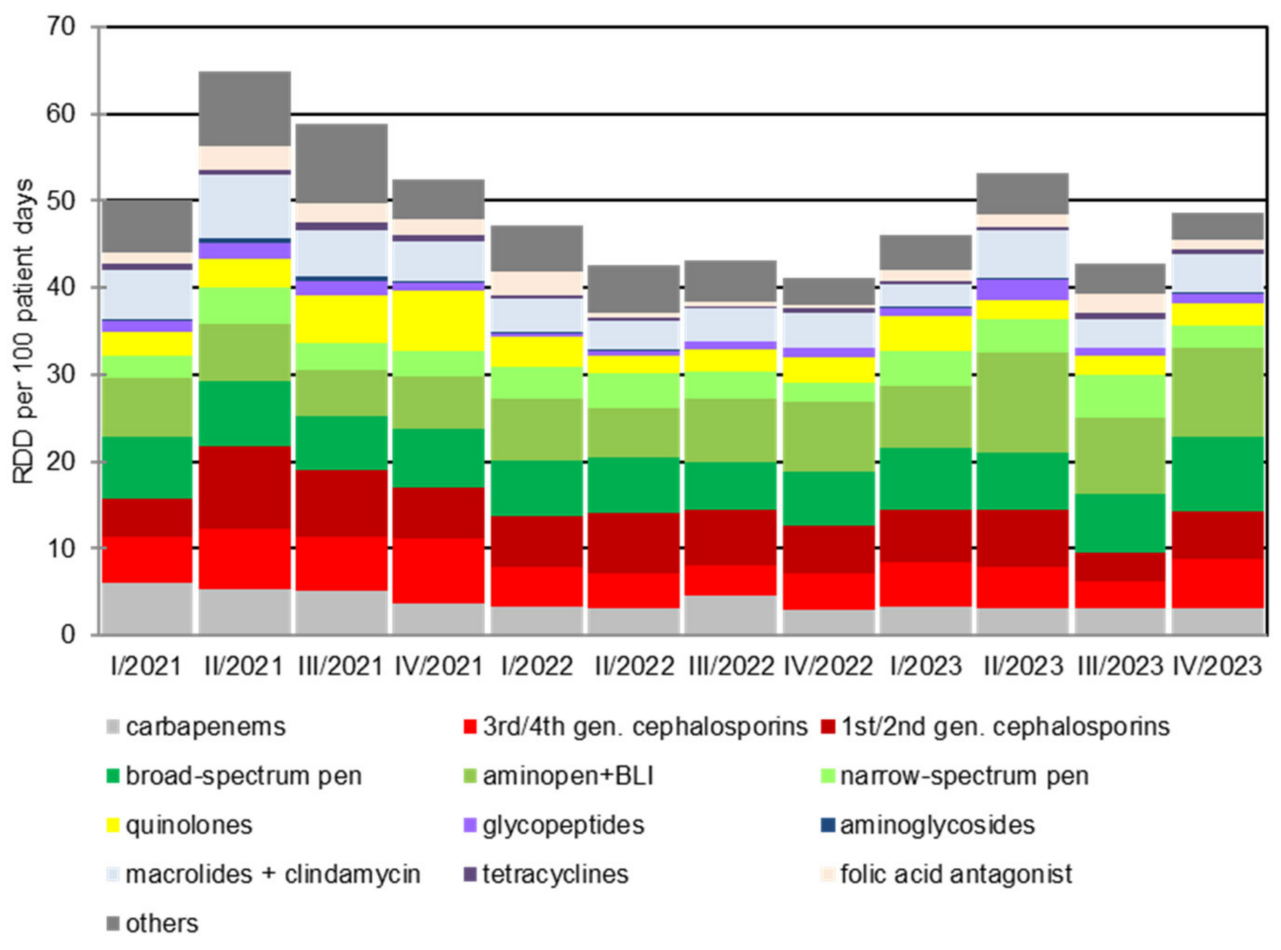
Development of antibiotic consumption density from the 1^st^ quarter of 2021 to the 4^th^ quarter of 2023. The implementation of the ABS consultation service at the Bundeswehr Central Hospital Koblenz took place in the 2^nd^ Quarter 2021. RDD: recommended daily dose; pen.: penicillin; gen.: generation; BLI: beta-lactamase inhibitor.

The development of antibiotic consumption from the 2^nd^ quarter of 2021 to the 4^th^ quarter of 2023 is shown in Figure 2 (Fig. 2). The retrograde development of antibiotic consumption density from the 2^nd^ quarter of 2021 is evident. The total consumption density per quarter fell statistically significantly (*p*=0.023) from 64.9 RDD/100 patient days to 42.5 RDD/100 patient days within 12 months up to the 2nd quarter of 2022. Despite the increasing number from the 1^st^ quarter of 2023, the consumption density after 24 months was still significantly lower than at the beginning of the observation period at 53.2 RDD/100 patient days (*p*=0.003). This result continued until the end of 2023. The retrograde development is also evident in comparison with other hospitals participating in the national project: In 2022, the consumption density of BwZKrhs Koblenz was in the middle of the comparison group (Figure 1B (Fig. 1)). 

### Trend development of selected antibiotic classes

The decline in carbapenem consumption is particularly noteworthy: it halved over the observation period from initially 5.3 RDD/100 patient days [95% CI 4.9–7.8] in the 2^nd^ quarter of 2021 to 3.0 RDD/100 patient days [95% CI 2.7–3.4] in the 4^th^ quarter of 2023 (*p*<0.001). A comparable trend can be observed for fluoroquinolones, where the consumption density fell from a maximum of 7.0 RDD/100 patient days [95% CI 6.5–7.6] in the 4^th^ quarter of 2021 to 2.5 RDD/100 patient days [95% CI 2.2–2.8] and was thus below the initial level of 3.3 RDD/100 [95% CI 3.0–3.7] patient days (*p*<0.001). This is in contrast to a progressive consumption density of beta-lactam antibiotics, especially penicillins. The consumption density of these increased significantly from 18.3 RDD/100 patient days [95% CI 17.5–19.2] in the 2^nd^ quarter of 2021 to 21.5 RDD/100 patient days [95% 20.6–22.4] in the 4^th^ quarter of 2023. Measured as a percentage of total consumption, a significant increase from 18.4% to 26.4% was observed in the same period (*p*=0.021). A slight upward trend in the prescription of narrow-spectrum and aminopenicillins was also observed.

### Germany-wide comparison

In the “ADKA-if-DGI” project 2019/2020, an antibiotic consumption density of 61 RDD/100 was determined for the BwZKrhs Koblenz. A total consumption density of 46 RDD/100 was calculated for 2022/2023. This corresponds to a reduction of 25% compared to 2019/2020 (Figure 2 (Fig. 2)).

### Need for counseling

**Figure 3:**
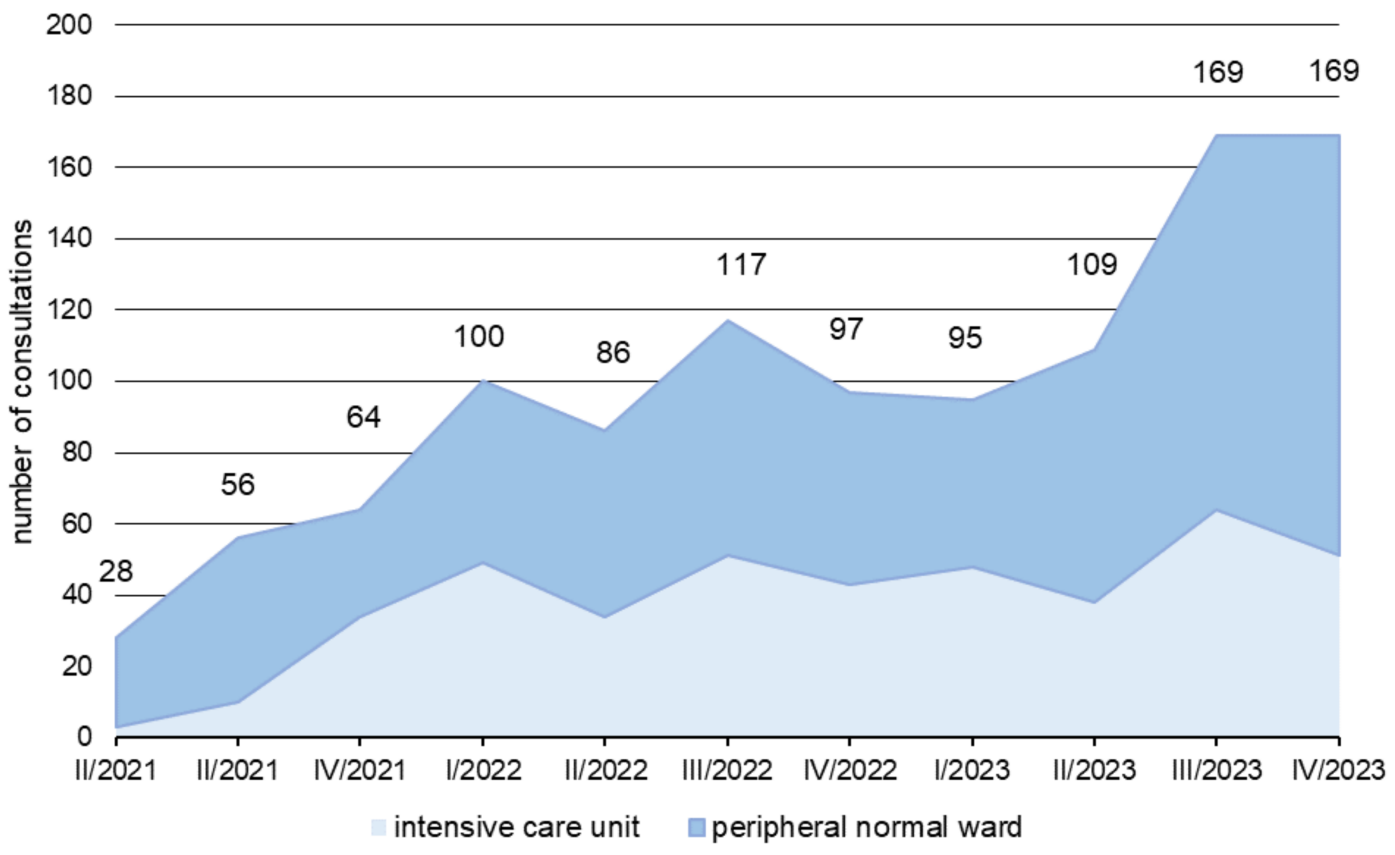
Development of the number of ABS consultations in the period from the 1^st^ quarter of 2022 to the 4^th^ quarter of 2023; the total number of all consultations in a quarter is shown with a colored representation of the proportion of consultations in peripheral normal wards and ICUs.

In the period from April 2021 to December 2023, 665 ABS consultations were carried out on peripheral wards, of which 84% (560/665) were within one working day. Over time, we saw an increase in ABS requests in the HIS. While a total of 101 consultation requests were received in 2021, by 2023 there were already 341 consultations (Figure 3 (Fig. 3)). This corresponds to an increase of 238%. In the period from April 2021 to December 2023, 425 cases were documented for the ABS ward-rounds service in the ICUs. A relatively constant number of cases were processed per quarter. There was a significant, strong inverse correlation between the total antibiotic consumption density, the carbapenem consumption density, and the number of consultations per quarter, respectively (r=–0.642 and r=–0.641, *p*=0.033).

## Discussion

The analysis of the quarterly antibiotic consumption density shows that the use of antibiotics has been declining since the measures presented above were introduced. The use of antibiotics is subject to natural fluctuations depending on the patient cohort and the clinical conditions that occur, but a clear trend from 2021 to 2023 is noticeable and statistically significant. 

Carbapenems in particular are being used in smaller quantities. This can be attributed to the efforts of the ABS team to promote the rational use of carbapenems. In the various samples analyzed by the Department of Medical Microbiology and Hospital Hygiene, no increased resistance was detected for Enterobacterales, so that in 2022, for example, only 8% of isolates of Escherichia coli and 7% of *Klebsiella* *pneumoniae* isolates were identified as resistant to 3^rd^ generation cephalosporins (e.g., cefotaxime and ceftazidime) (unpublished data of the current authors). In *Pseudomonas* *aeruginosa*, 17% of isolates tested as resistant to piperacillin-tazobactam and 10% as sensitive to ciprofloxacin (unpublished data of the current authors). Thus, there was no conclusive justification for the excessive carbapenem use to date. In patients without colonization or infection with MDR bacteria, the options for de-escalation in the sense of “carbapenem sparing” [12] are always indicated. The use of broad-spectrum penicillins or 3^rd^/4^th^ generation cephalosporins may represent alternative treatment options, depending on the infection [12], [13], [14]. If resistance testing is available, corresponding recommendations for a change in therapy are given to minimize the untargeted use of reserve antibiotics, e.g., carbapenems. The preferred prescription of penicillins over cephalosporins is assessed as favourable regarding the risk of adverse drug reactions. The changes are particularly clear in comparison with other hospitals with a similar number of beds: while the BwZKrhs was still the top consumer in its peer group in 2020, it was in the lower midfield in 2022.

## Conclusion

The results indicate that the implementation and consolidation of ABS measures not only has a positive but also a direct effect on antibiotic prescribing behavior at Koblenz Central Hospital. However, the effect presented here cannot be attributed solely to the ABS consultation and ward-rounds service; other ABS measures, such as therapeutic drug monitoring (TDM) and the restriction of reserve antibiotics, have also been implemented at BwZKrhs Koblenz, meaning that the positive development of antibiotic prescribing behavior must be considered in the context of the entire package of interventions. Similar results can be found in both national [15], [16], [17] and international data [18], [19], [20].

The distribution of reserve anti-infectives as well as TDM by the hospital pharmacy are subjects to validation by an ABS team member, so that corresponding requests serve the ABS team as an indicator for complicated infections. The validation process therefore makes it possible to proactively offer counseling to the treating physicians. In addition to individual therapy recommendations, the work of the ABS team as part of the consultation and ward-rounds service also serves to provide information on the rational use of antibiotics. This not only increases the acceptance of ABS measures, but also promotes the responsible use of anti-infectives in the long term. Patient-specific dose optimization as part of TDM avoids unnecessarily high doses and increases the safety of drug therapy for patients by reducing adverse drug reactions.

ABS lives, works, and flourishes through interdisciplinary cooperation and combines the expertise of different professions. The joint coordinated approach offers the opportunity for a mutual exchange of experience and ensures greater acceptance of the implemented measures. Personal dialogue with treating physicians in particular provides a valuable opportunity to further increase awareness of the rational use of antibiotics. The implementation of ABS thus leads to more than just a reduction in antibiotic consumption as the sum of individual measures. 

An interdisciplinary ABS approach can help to ensure the effectiveness of anti-infective therapies for current and future patients.

## Notes

### Authors’ ORCID

- Gwendolyn Scheumann: 0009-0000-4470-346X
- Manuel Döhla: 0000-0001-8029-5264
- Dominic Rauschning: 0000-0001-9284-8778

### Funding 

None.

### Competing interests

The authors declare that they have no competing interests.

## References

[1] O’Neill J (2014). Antimicrobial Resistance: Tackling a Crisis for the Health and Wealth of Nations.

[2] Antimicrobial Resistance Collaborators (2022). Global burden of bacterial antimicrobial resistance in 2019: a systematic analysis. Lancet.

[3] European Centre for Disease Prevention and Control (2022). Health burden of infections with antibiotic-resistant bacteria in the EU/EEA, 2016-2020.

[4] Word Health Organization (2022). 2021 Antibacterial agents in clinical and preclinical development: an overview and analysis. https://www.who.int/publications/i/item/9789240047655.

[5] Shafiq N, Pandey AK, Malhotra S, Holmes A, Mendelson M, Malpani R, Balasegaram M, Charani E (2021). Shortage of essential antimicrobials: a major challenge to global health security. BMJ Glob Health.

[6] Huss G, Barak S, Reali L, Magendie C, Carrasco-Sanz A, Somekh E, Cohen R, Levy C, Namazova-Baranova L, Vural M, Pettoello-Mantovani M (2023). Drug Shortages in Pediatrics in Europe: The Position of the European Pediatric Societies. J Pediatr.

[7] World Health Organization (2024). WHO bacterial priority pathogens list, 2024: Bacterial pathogens of public health importance to guide research, development and strategies to prevent and control antimicrobial resistance.

[8] Gesetz zur Verhütung und Bekämpfung von Infektionskrankheiten beim Menschen: Infektionsschutzgesetz (IfSG). Infektionsschutzgesetz vom 20. Juli 2000 (BGBl. I S. 1045), zuletzt durch Artikel 8v des Gesetzes vom 12. Dezember 2023 (BGBl. 2023 I Nr. 359) geändert.

[9] Deutsche Gesellschaft für Infektiologie e.V. (2013). S3 Leitlinie Strategien zur Sicherung rationaler Antibiotika Anwendung im Krankenhaus. AWMF-Registernummer 092/001 – update 2018.

[10] ADKA, if, dgi Antiinfektiva Surveillance.

[11] Robert-Koch-Institut Die Surveillanceprojekte.

[12] Corcione S, Lupia T, Maraolo AE, Mornese Pinna S, Gentile I, De Rosa FG (2019). Carbapenem-sparing strategy: carbapenemase, treatment, and stewardship. Curr Opin Infect Dis.

[13] Cao J, Dubrovskaya Y, Siegfried J, Decano A, Mazo D, Hochman S, Zacharioudakis IM, So J, Solomon S, Papadopoulos J, Marsh K (2023). Treatment of Piperacillin-Tazobactam-Nonsusceptible/Ceftriaxone-Susceptible Infections With Carbapenem Versus Carbapenem-Sparing Antimicrobials. Open Forum Infect Dis.

[14] Pilmis B, Jullien V, Tabah A, Zahar JR, Brun-Buisson C (2017). Piperacillin-tazobactam as alternative to carbapenems for ICU patients. Ann Intensive Care.

[15] Feihl S, Querbach C, Hapfelmeier A, Busch DH, von Eisenhart-Rothe R, Gebhardt F, Pohlig F, Mühlhofer HML (2022). Effect of an Intensified Antibiotic Stewardship Program at an Orthopedic Surgery Department. Surg Infect (Larchmt).

[16] Gruber MM, Weber A, Jung J, Werner J, Draenert R (2021). Impact and Sustainability of Antibiotic Stewardship on Antibiotic Prescribing in Visceral Surgery. Antibiotics (Basel).

[17] Schröder S, Klein MK, Heising B, Lemmen SW (2020). Sustainable implementation of antibiotic stewardship on a surgical intensive care unit evaluated over a 10-year period. Infection.

[18] Garcell HG, Arias AV, Fernandez EA, Guerrero YB, Serrano RN (2016). Antibiotic Consumption During a 4-year Period in a Community Hospital with an Antimicrobial Stewardship Program. Oman Med J.

[19] Lee CF, Cowling BJ, Feng S, Aso H, Wu P, Fukuda K, Seto WH (2018). Impact of antibiotic stewardship programmes in Asia: a systematic review and meta-analysis. J Antimicrob Chemother.

[20] Mahmoudi L, Sepasian A, Firouzabadi D, Akbari A (2020). The Impact of an Antibiotic Stewardship Program on the Consumption of Specific Antimicrobials and Their Cost Burden: A Hospital-wide Intervention. Risk Manag Healthc Policy.

